# Sudden Cardiac Death in Athletes: Consensuses and Controversies

**DOI:** 10.7759/cureus.39873

**Published:** 2023-06-02

**Authors:** Usman Ghani, Omer Farooq, Sundus Alam, Muhammad Junaid Khan, Omar Rahim, Sarah Rahim

**Affiliations:** 1 Cardiology, Northwest General Hospital and Research Center, Peshawar, PAK; 2 Internal Medicine, Presence Saint Francis Hospital, Evanston, USA; 3 Cardiology, Gloucestershire Hospitals NHS Foundation Trust, Gloucester, GBR; 4 Orthopaedic Surgery, Gloucestershire Hospitals NHS Foundation Trust, Gloucester, GBR; 5 Internal Medicine, Naseer Teaching Hospital, Peshawar, PAK; 6 Cardiology, Rehman Medical Institute, Peshawar, PAK

**Keywords:** mechanism, prevention, cause, incidence, athlete's heart, athlete, sudden cardiac death (scd)

## Abstract

Exercise is widely considered beneficial for cardiovascular health. However, on rare occasions, athletes experience sudden cardiac death without any preceding symptoms. The devastating nature of these events necessitates us to understand the underlying causes. In younger athletes (age <35), the underlying causes are usually hereditary/genetic, whereas in older athletes (age >35), coronary artery disease is prevalent. Sudden cardiac death in athletes can occur regardless of the presence of any structural abnormality in the heart. Despite divergence between guidelines, the majority of cardiology societies recommend at least taking a comprehensive history and performing physical examinations for initial screening for all athletes. This article reviews the consensuses and controversies regarding the incidence, causes, and prevention of sudden cardiac death in athletes.

## Introduction and background

Generally, sudden cardiac death in athletes is death occurring from cardiac arrest within an hour of acute change in clinical status if the event was witnessed, or an unexpected death within 24 hours if the episode was not witnessed. The reported incidence of sudden cardiac death in athletes has been highly variable across studies. We explore the methodical variations across studies that have led to these inconsistencies. Moreover, the consensus on certain groups of athletes who are relatively at a higher risk is also discussed.

Sudden cardiac death usually presents in athletes without any previously known cardiac conditions who are otherwise asymptomatic. The demanding nature of competitive sports can precipitate a cardiac arrest in the presence of an underlying cardiac pathology. These catastrophic events are usually the first presentation of the underlying condition. Screening for these conditions can prevent sudden cardiac death but there is an ongoing debate as to which modalities should be included and whether widespread screening should even be reinforced as athletes may be disqualified from participating in the sport [1].

Sudden cardiac death in athletes is caused by both genetic/congenital and acquired cardiac conditions. Both groups of athletes (with structurally normal or abnormal hearts) are at risk. These athletes are usually asymptomatic until the event. Sometimes (in athletes with structurally normal hearts) the underlying condition is not identified even on autopsy. Sudden cardiac death in older athletes (age >35) is usually due to underlying coronary artery disease, however, in younger athletes (age <35), ion channelopathies and primary cardiomyopathies are usually the underlying causes [2].

Continual research is needed to understand the underlying causes of sudden cardiac death in athletes, with the ultimate goal of devising preventive strategies to reduce the incidence in the population.

Methods

A review of the literature was done from electronic databases (Pubmed, Scopus, and Google Scholar) to identify relevant peer-reviewed articles. Publications from 1959 to 2023 were included. The following search terms were used, “sudden cardiac death’’ and “athlete”. Articles in the English language were included. The inclusion criteria were not exclusive to any specific study design. For this study, 283 relevant articles were included and 50 were referenced. Findings were presented using a narrative approach. The data were summarised to highlight consensuses and controversies regarding the incidence, causes, and prevention of sudden cardiac death in athletes.

## Review

Differentiating pathologic cardiac conditions from athlete's heart

Preventing sudden cardiac death in athletes includes careful identification of the underlying cardiac pathologies. Therefore, it is imperative to understand the differences between normal physiologic and pathologic changes in the heart [3,4]. Table 1 delineates the physiologic changes that occur in athlete’s heart.

**Table 1:** Physiologic changes in athlete's heart AV- Atrioventricular; LVH- left ventricular hypertrophy; RVH- right ventricular hypertrophy; RBBB- right bundle branch block

Electrical changes	Structural changes	Functional changes
Sinus bradycardia	Increased left and right ventricle cavity	Increased diastolic filling
Sinus arrhythmia	Increased left ventricular wall thickness	Increased augmentation of stroke volume
First degree AV block	Biatrial enlargement	
Voltage criteria LVH and RVH		
Incomplete RBBB		

Cardiac adaptations can vary depending on the type of exercise athletes perform. Table 2 differentiates between the cardiac changes that occur in athletes based on the type of training they undergo while also indicating the similarities.

**Table 2:** Physiologic changes due to different types of activities LV- left ventricle; LA- left atrium; LVWT- left ventricular wall thickness; RV- right ventricle

Resistance Training	Aerobics
Concentric LV hypertrophy	Eccentric LV hypertrophy
Mild LA hypertrophy. Increased LVWT and minimal change in LV volume	LV dilation and proportional LVWT
Mild LA hypertrophy	Mild RV dilation
Activation of progenitor cells	Biatrial enlargement
Reversible	Cardiomyocyte proliferation
	Activation of progenitor cells
	Reversible

Cardiac physiologic adaptations are usually mild/moderate and are reversible, however, pathologic changes are usually severe and irreversible. The major differences in physiologic and pathologic changes in the heart are mentioned in Table 3.

**Table 3:** Common differences in cardiac physiologic and pathologic hypertrophy SCD- sudden cardiac death; HF- heart failure

Physiologic hypertrophy	Pathologic hypertrophy
Reversible	Irreversible
Low incidence of SCD and HF	High incidence of SCD and HF
Regression with detraining	Does not regress
Mild/moderate structural changes	Frank structural changes
Normal cardiac function	Reduced cardiac function

Incidence of sudden cardiac death in athletes

There is huge variability in the reported incidence of sudden cardiac death in athletes across numerous studies. Steinvil et al. [5] reported an incidence of 1/39000 (per athlete-person years), whereas, Van Camp et al. [6] reported an incidence of 1/281,000 (per athlete-person years). Due to the lack of a unifying definition of an athlete, the definition of sudden cardiac death in an athlete varies, which leads to heterogeneous grouping across studies and selection bias. Some studies include exclusively sudden cardiac deaths [7-10], while others have broader inclusive criteria that include sudden cardiac deaths and sudden cardiac arrests (reverted with the return of spontaneous circulation) [11,12]. Moreover, there is a lack of similar enforcement of screening and provision of automated external defibrillators in different countries which leads to inconsistent findings across studies carried out in different geographic locations [2]. The lack of a thorough post-mortem evaluation by cardiac pathologists or even a thorough evaluation in patients with structurally normal hearts may also lead to inaccurate diagnosis of the cause of death.

There appears to be a general consensus that the risk of sudden cardiac death in athletes increases with age [13-16]. The incidence of sudden cardiac death in athletes is greater in certain groups, namely, male [10,15,17-23], african american players [10,17-19], and basketball players [17,19,24]. However, studies carried out in Spain [25] and Italy [21] reported a relatively higher incidence among soccer players. This may be due to the fact that more athletes in Europe play soccer.

Causes of sudden cardiac death in athletes

Sudden cardiac death in athletes is caused by a variety of conditions. In older athletes (age >35), the most common cause is usually reported to be coronary artery disease [26]. It is still up for debate whether the sudden cardiac death is due to plaque rupture or due to the imbalance in supply and demand [19].

In younger athletes (age <35), the most common pathway for sudden cardiac death is an arrhythmia which may be precipitated by different underlying pathologies. Hypertrophic cardiomyopathy is often reported as the most common underlying cause in this age group [26], however, studies in Italy [27] and Denmark [28] have reported the most common underlying cause to be arrhythmogenic right ventricular cardiomyopathy. An analysis of 1866 cases reported congenital coronary artery anomalies to be the second most common cause [29].

The various causes of sudden cardiac death in athletes are presented in Table 4.

**Table 4:** Common cardiac conditions causing sudden cardiac death in athletes ARVC- Arrhythmogenic right ventricular cardiomyopathy; WPW- Wolff-Parkinson-White; CPVT- Catecholaminergic polymorphic ventricular tachycardia

Structurally Abnormal Heart	Structurally Normal Heart
Hypertrophic cardiomyopathy	Substance ingestion
Dilated cardiomyopathy	WPW syndrome
Coronary artery disease (athletes >35 years old)	Commotio Cordis
ARVC	Brugada syndrome
Valvular heart disease	Long QT syndrome
Congenital abnormalities of the coronary arteries	Electrolyte abnormalities
Marfan syndrome	CPVT
Myocarditis	Environmental factors
Left ventricular non-compaction cardiomyopathy	Substance ingestion
Kawasaki disease	

Mechanisms of sudden cardiac death in athletes

The aforementioned causes lead to sudden cardiac death in athletes through the following mechanisms.

Coronary Artery Disease

Coronary artery disease causes sudden cardiac death [26] either due to plaque rupture or due to the incapacity of the myocardium to adapt to increased requirements of the body during exertion [19].

Hypertrophic Cardiomyopathy, Dilated Cardiomyopathy, Arrhythmogenic Right Ventricular Cardiomyopathy, Electrolyte Abnormalities, Long QT Syndrome, Brugada Syndrome, and Myocarditis

Hypertrophic cardiomyopathy [30], dilated cardiomyopathy [31], electrolyte abnormalities [32], myocarditis [30], long QT syndrome (a common cause of sudden cardiac death in swimmers) [33], Brugada syndrome [34], and arrhythmogenic right ventricular cardiomyopathy [28,30] can precipitate ventricular tachycardia/ventricular fibrillation, ultimately leading to sudden cardiac death.

Coronary Artery Anomalies

The different types of coronary artery anomalies can lead to myocardial ischemia and sudden cardiac death in the setting of increased physical activity [30,34].

Catecholaminergic Polymorphic Ventricular Tachycardia

Catecholaminergic polymorphic ventricular tachycardia, as evidenced by the name, causes ventricular tachycardia (premature ventricular beats) [30].

Left Ventricular Non-compaction Cardiomyopathy

Left ventricular non-compaction cardiomyopathy causes heart failure and ventricular arrhythmia [35].

Wolff-Parkinson-White Syndrome

Wolff-Parkinson-White syndrome causes sudden cardiac death usually through an arrhythmia (atrial fibrillation/ventricular fibrillation) [36].

Valvular Heart Disease

Valvular heart disease leads to an imbalance in supply and demand either due to stenosis or insufficiency [37].

Marfan Syndrome

Marfan syndrome causes sudden cardiac death through aortic root dissection or rupture [34].

Kawasaki Disease

Coronary artery aneurysms in patients with Kawasaki disease can cause stenosis and occlusion of the coronary artery due to aneurysm remodeling, thereby causing myocardial ischemia [38].

Commotio Cordis

Commotio cordis causes ventricular fibrillation and cardiac arrest in normal hearts (precipitated by trauma) [34].

Performance-Enhancing Substances

Performance-enhancing substances can cause sudden cardiac death through different mechanisms depending on the specific substance being taken. Energy drinks/caffeine cause increased demand and reduced supply thereby causing myocardial ischemia leading to ventricular arrhythmia [39]. Anabolic steroids cause hypertension, dyslipidemia, myocyte hypertrophy, interstitial fibrosis, cardiac remodeling, and exercise-induced cardiac injury. There have been proposed four different mechanisms leading to sudden cardiac death in anabolic steroid users (atherogenic, thrombosis, vasospasm, or direct myocardial injury) [40]. Erythropoietin increases the risk of thrombosis [41] and growth hormones cause cardiac remodeling [42].

Hypothermia and Heatstroke

Hypothermia reduces pacemaker automaticity and disturbs ion channel activity eventually leading to reduced cardiac output, it also causes coronary ischemia and arrhythmias (tachycardia and atrial fibrillation). Heatstroke causes heart failure and fatal cardiac arrest.

Preventive strategies

Prevention of sudden cardiac death in athletes can be achieved through pre-participation screening, training in performing cardiopulmonary resuscitation, provision of automated external defibrillators, and team education.

Pre-participation Screening

There is no general consensus on what should be included in the screening and whether screening should even be implemented. American Heart Association, American College of Cardiology and European Society of Cardiology recommend taking a detailed history along with performing a physical examination [43-47], however, American Heart Association does not recommend electrocardiography, whereas, the European Society of Cardiology recommends it as an additional screening tool [44-46]. Some factors that encourage the decision to not recommend electrocardiography are false negative and false positive results, uncertain benefits, and cost-related issues. Disqualification of the athlete from the sport is a concern that leads to recommendations against screening altogether [1]. Any concerning findings on the above-mentioned evaluations should be followed by further relevant investigations like echocardiography, cardiac stress test, 24-hour Holter monitoring, and cardiac magnetic resonance imaging [48].

Cardiopulmonary Resuscitation and Automated External Defibrillators

Cardiopulmonary resuscitation and automated external defibrillators can be lifesaving in the setting of an unprecedented cardiac arrest. Certain cases of sudden cardiac death in athletes occur in a normal heart (due to commotio cordis), which cannot be prevented by screening and must be resuscitated with cardiopulmonary resuscitation and automated external defibrillators.

Team Education

As mentioned in Table 4, substance ingestion can cause sudden cardiac death, therefore, it is crucial to encourage athletes to discuss with a doctor the risks and benefits of any performance-enhancing substances they might be taking. Athletes should also be careful to avoid hypothermia and heat stroke. Any symptoms like chest pain, palpitations, and shortness of breath warrant an evaluation by a doctor.

Previously, younger athletes with channelopathies were advised to refrain from sports entirely, but with our current understanding of the mechanisms, improved anti-arrhythmic medications, and larger available data, American Heart Association and American College of Cardiology guidelines are more lenient, thereby allowing certain patients with long QT syndrome to participate in sports provided they get regular checkups and take appropriate medications [49,50].

## Conclusions

In light of the high variability of reported incidence of sudden cardiac death in athletes, we recommend further studies be done. Any concerning symptoms (chest pain, shortness of breath, palpitations) experienced by athletes warrant an evaluation by a doctor. Pre-participation screening should be a shared decision due to concerns regarding false positive/false negative results, cost-effectiveness, and psychological impact on athletes who may be disqualified from the sport. Pre-participation screening should include a detailed history, followed by a thorough physical examination (electrocardiography is also advisable based on European guidelines). Any concerning findings on pre-participation screening warrant further relevant investigations (echocardiography, cardiac stress test, 24-hour Holter monitoring, and cardiac magnetic resonance imaging). Moreover, it would be sensible to keep a lower threshold for pre-participation screening in certain groups of athletes who are relatively at a higher risk of sudden cardiac death (age >35, men, African American athletes, basketball players, and soccer players in Europe). As sudden cardiac death in athletes cannot always be prevented even through the implementation of widespread screening, educating athletes about cardiac symptoms, the effects of substance ingestion, and lifestyle modifications are imperative. Lastly, training in cardiopulmonary resuscitation and the provision of automated external defibrillators can significantly reduce the incidence.

## References

[1] Magavern EF, Finocchiaro G, Sharma S, Papadakis M, Borry P (2018). Time out: ethical reflections on medical disqualification of athletes in the context of mandated pre-participation cardiac screening. Br J Sports Med.

[2] Han J, Lalario A, Merro E, Sinagra G, Sharma S, Papadakis M, Finocchiaro G (2023). Sudden cardiac death in athletes: facts and fallacies. J Cardiovasc Dev Dis.

[3] Sharma S, Drezner JA, Baggish A (2018). International recommendations for electrocardiographic interpretation in athletes. Eur Heart J.

[4] Sanz-de la Garza M, Carro A, Caselli S (2020). How to interpret right ventricular remodeling in athletes. Clin Cardiol.

[5] Steinvil A, Chundadze T, Zeltser D (2011). Mandatory electrocardiographic screening of athletes to reduce their risk for sudden death proven fact or wishful thinking?. J Am Coll Cardiol.

[6] Van Camp SP, Bloor CM, Mueller FO, Cantu RC, Olson HG (1995). Nontraumatic sports death in high school and college athletes. Med Sci Sports Exerc.

[7] Drezner JA, Rogers KJ, Zimmer RR, Sennett BJ (2005). Use of automated external defibrillators at NCAA Division I universities. Med Sci Sports Exerc.

[8] Harmon KG, Asif IM, Klossner D, Drezner JA (2011). Incidence of sudden cardiac death in National Collegiate Athletic Association athletes. Circulation.

[9] Maron BJ, Haas TS, Murphy CJ, Ahluwalia A, Rutten-Ramos S (2014). Incidence and causes of sudden death in U.S. college athletes. J Am Coll Cardiol.

[10] Harmon KG, Asif IM, Maleszewski JJ (2015). Incidence, etiology, and comparative frequency of sudden cardiac death in NCAA athletes: a decade in review. Circulation.

[11] Drezner JA, Rao AL, Heistand J, Bloomingdale MK, Harmon KG (2009). Effectiveness of emergency response planning for sudden cardiac arrest in United States high schools with automated external defibrillators. Circulation.

[12] Toresdahl BG, Rao AL, Harmon KG, Drezner JA (2014). Incidence of sudden cardiac arrest in high school student athletes on school campus. Heart Rhythm.

[13] Chugh SS, Weiss JB (2015). Sudden cardiac death in the older athlete. J Am Coll Cardiol.

[14] Narayanan K, Bougouin W, Sharifzadehgan A, Waldmann V, Karam N, Marijon E, Jouven X (2017). Sudden cardiac death during sports activities in the general population. Card Electrophysiol Clin.

[15] Couper K, Putt O, Field R (2020). Incidence of sudden cardiac death in the young: a systematic review. BMJ Open.

[16] Risgaard B (2016). Sudden cardiac death: a nationwide cohort study among the young. Dan Med J.

[17] Asif IM, Harmon KG (2017). Incidence and etiology of sudden cardiac death: new updates for athletic departments. Sports Health.

[18] Sealy DP, Pekarek L, Russ D, Sealy C, Goforth G (2010). Vital signs and demographics in the preparticipation sports exam: do they help us find the elusive athlete at risk for sudden cardiac death?. Curr Sports Med Rep.

[19] Wasfy MM, Hutter AM, Weiner RB (2016). Sudden cardiac death in athletes. Methodist Debakey Cardiovasc J.

[20] Suzuki-Yamanaka M, Ayusawa M, Hosokawa Y, Hirose N, Kaneoka K (2022). Epidemiology of sudden cardiac death and sudden cardiac arrest with resultant disability during high school organized sport in Japan. J Sci Med Sport.

[21] Sollazzo F, Palmieri V, Gervasi SF (2021). Sudden cardiac death in athletes in Italy during 2019: internet-based epidemiological research. Medicina (Kaunas).

[22] Rücklová K, Dobiáš M, Bílek M (2022). Burden of sudden cardiac death in persons aged 1-40 years in the Czech Republic. Cent Eur J Public Health.

[23] Chappex N, Schlaepfer J, Fellmann F, Bhuiyan ZA, Wilhelm M, Michaud K (2015). Sudden cardiac death among general population and sport related population in forensic experience. J Forensic Leg Med.

[24] Harmon KG, Drezner JA, Wilson MG, Sharma S (2014). Incidence of sudden cardiac death in athletes: a state-of-the-art review. Br J Sports Med.

[25] Morentin B, Suárez-Mier MP, Monzó A, Ballesteros J, Molina P, Lucena J (2021). Sports-related sudden cardiac death in Spain. A multicenter, population-based, forensic study of 288 cases. Rev Esp Cardiol (Engl Ed).

[26] Emery MS, Kovacs RJ (2018). Sudden cardiac death in athletes. JACC Heart Fail.

[27] Holst AG, Winkel BG, Theilade J (2010). Incidence and etiology of sports-related sudden cardiac death in Denmark--implications for preparticipation screening. Heart Rhythm.

[28] Corrado D, Basso C, Rizzoli G, Schiavon M, Thiene G (2003). Does sports activity enhance the risk of sudden death in adolescents and young adults?. J Am Coll Cardiol.

[29] Maron BJ, Doerer JJ, Haas TS, Tierney DM, Mueller FO (2009). Sudden deaths in young competitive athletes: analysis of 1866 deaths in the United States, 1980-2006. Circulation.

[30] Vora A, Burkule N, Contractor A, Bhargava K (2018). Prevention of sudden cardiac death in athletes, sportspersons and marathoners in India. Indian Heart J.

[31] Wu AH, Das SK (1999). Sudden death in dilated cardiomyopathy. Clin Cardiol.

[32] American Heart Association (2005). Part 10.1: life-threatening electrolyte abnormalities. Circulation.

[33] Schwartz PJ, Priori SG, Spazzolini C (2001). Genotype-phenotype correlation in the long-QT syndrome: gene-specific triggers for life-threatening arrhythmias. Circulation.

[34] Chandra N, Bastiaenen R, Papadakis M, Sharma S (2013). Sudden cardiac death in young athletes: practical challenges and diagnostic dilemmas. J Am Coll Cardiol.

[35] Kamalov G (2015). The risk of sudden cardiac death in a patient with non-compaction cardiomyopathy. EP Lab Dig.

[36] Timmermans C, Smeets JL, Rodriguez LM, Vrouchos G, van den Dool A, Wellens HJ (1995). Aborted sudden death in the Wolff-Parkinson-White syndrome. Am J Cardiol.

[37] Henriques de Gouveia RH, Corte Real Gonçalves FM (2019). Sudden cardiac death and valvular pathology. Forensic Sci Res.

[38] Zhu KF, Tang LJ, Wu SZ, Tang YM (2019). Out-of-hospital cardiac arrest in a young adult survivor with sequelae of childhood Kawasaki disease: A case report. World J Clin Cases.

[39] Higgins JP, Babu KM, Deuster PA, Shearer J (2018). Stimulant-containing energy drinks. ACSMs Health Fit J.

[40] Torrisi M, Pennisi G, Russo I (2020). Sudden cardiac death in anabolic-androgenic steroid users: a literature review. Medicina (Kaunas).

[41] Smith KJ, Bleyer AJ, Little WC, Sane DC (2003). The cardiovascular effects of erythropoietin. Cardiovasc Res.

[42] Maison P, Chanson P (2003). Cardiac effects of growth hormone in adults with growth hormone deficiency: a meta-analysis. Circulation.

[43] Bagnall RD, Weintraub RG, Ingles J (2016). A prospective study of sudden cardiac death among children and young adults. N Engl J Med.

[44] Pelliccia A, Sharma S, Gati S (2021). 2020 ESC Guidelines on sports cardiology and exercise in patients with cardiovascular disease. Eur Heart J.

[45] Corrado D, Pelliccia A, Bjørnstad HH (2005). Cardiovascular pre-participation screening of young competitive athletes for prevention of sudden death: proposal for a common European protocol. Consensus Statement of the Study Group of Sport Cardiology of the Working Group of Cardiac Rehabilitation and Exercise Physiology and the Working Group of Myocardial and Pericardial Diseases of the European Society of Cardiology. Eur Heart J.

[46] Maron BJ, Levine BD, Washington RL, Baggish AL, Kovacs RJ, Maron MS (2015). Eligibility and disqualification recommendations for competitive athletes with cardiovascular abnormalities: task force 2: preparticipation screening for cardiovascular disease in competitive athletes: a scientific statement from the American Heart Association and American College of Cardiology. Circulation.

[47] Drezner JA, OʼConnor FG, Harmon KG (2016). AMSSM position statement on cardiovascular preparticipation screening in athletes: current evidence, knowledge gaps, recommendations and future directions. Clin J Sport Med.

[48] Mavrogeni SI, Bacopoulou F, Apostolaki D, Chrousos GP (2018). Sudden cardiac death in athletes and the value of cardiovascular magnetic resonance. Eur J Clin Invest.

[49] Lang CN, Steinfurt J, Odening KE (2017). Avoiding sports-related sudden cardiac death in children with congenital channelopathy : Recommendations for sports activities. Herz.

[50] Schnell F, Behar N, Carré F (2018). Long-QT syndrome and competitive sports. Arrhythm Electrophysiol Rev.

